# Plant Disease Sensing: Studying Plant-Pathogen Interactions at Scale

**DOI:** 10.1128/mSystems.01228-21

**Published:** 2021-11-16

**Authors:** Kaitlin M. Gold

**Affiliations:** a Plant Pathology and Plant-Microbe Biology Section, School of Integrative Plant Sciences, Cornell AgriTech, Cornell University, Geneva, New York, USA

**Keywords:** agricultural microbiology, agriculture, host-microbe interactions, hyperspectral imaging, plant disease sensing, plant pathology, remote sensing

## Abstract

Plant disease threatens the environmental and financial sustainability of crop production, causing $220 billion in annual losses. The dire threat disease poses to modern agriculture demands tools for better detection and monitoring to prevent crop loss and input waste. The nascent discipline of plant disease sensing, or the science of using proximal and/or remote sensing to detect and diagnose disease, offers great promise to extend monitoring to previously unachievable resolutions, a basis to construct multiscale surveillance networks for early warning, alert, and response at low latency, an opportunity to mitigate loss while optimizing protection, and a dynamic new dimension to agricultural systems biology. Despite its revolutionary potential, plant disease sensing remains an underdeveloped discipline, with challenges facing both fundamental study and field application. This article offers a perspective on the current state and future of plant disease sensing, highlights remaining gaps to be filled, and presents a bold vision for the future of global agriculture.

## COMMENTARY

Plant disease changes how solar radiation interacts with leaves, canopy, and plant energy balance, which can be quantified with *in situ* and imaging spectroscopy. Also known as “hyperspectral imaging,” these tools are capable of early, non-destructive, and scalable biotic stress detection in both natural ecosystems and agroecosystems (1–5). Spectral data can allow one to draw inferences about vegetation health because of the relationship between plant chemical, physiological, anatomical, and morphological properties and light absorption at specific wavelengths. In terrestrial remote sensing, the idea of “foliar functional traits” has emerged as a unifying concept to better understand both natural variability in vegetation function and variability in the responses to stress (6). Many traits shown to strongly correlate with changes to plant function (7) can be accurately quantified and mapped with imaging spectroscopy (8–13). Originating in terrestrial ecology, this use of spectroscopy combined with physiochemistry and taxonomy has been called “spectranomics,” (10, 14). The foundational components of the spectranomics approach are that plants have chemical fingerprints that become increasingly unique when additional constituents are incorporated (9) and spectroscopic signatures determine a portfolio of chemicals found in plants (15). This approach has since been extended to phylogenetics (16, 17), conservation biology (14), and plant metabolic functioning (18) with great success. Plant pathologists have recently begun to take advantage of the spectranomics trail blazed by terrestrial ecologists, yielding the nascent discipline of plant disease sensing (17). Both beneficial (19) and parasitic (20) plant-microbe interactions impact a variety of plant traits that can be sensed. Plant pathogens damage, impair, and/or alter plant function, thus changing foliar composition, by such mechanisms as production of systemic effectors or secondary metabolites or by the physical presence of pathogen structures, such as hyphae and spores (21). Abiotic and biotic stresses have divergent spectral pathways, which is why spectroscopy can be used to differentiate between them (73). Broadband and multispectral methods relying on visible (Vis) and near-infrared (NIR) reflectance indices, such as the normalized difference vegetation index (NDVI), have been used to sense late-stage plant disease since the 1980s (22, 23). Changes in continuous, short-wave infrared (SWIR) wavelengths have proved valuable for plant disease sensing due to their sensitivity to a range of foliar properties (24), including nutrient content (12, 13, 25–28), water (29), photosynthetic capacity (30), physiology (31), phenolics (32), and secondary metabolites (18, 33) all impacted by early-stage disease. By taking a statistical approach, ranging from nonparametric statistics to deep learning, to hyperspectral data analysis (15), the sum total of direct and indirect changes that microbes impart to plant health can be quantified with proximal and remote spectroscopy (1, 2). Plant disease sensing allows us to detect, map, and model the biochemical and physiological pathosystem processes that underlie the diseased plant phenotype, thus forming the basis of our ability to use sensing for disease detection in the first place (4, 5, 34–40).

Plant disease sensing has great appeal due to its inherent scalability and capacity for passive monitoring. Most detection methods, spanning molecular assays to smartphone apps, require active sampling: a human must first seek out and observe disease conditions. Scouting, i.e., the well-trained human eye actively looking for disease in the field, has been the most widely relied-upon monitoring method since the dawn of agriculture. Heavy reliance on scouting for initial detection has unfortunately made management into a perpetual game of cat and mouse. The scale of modern agriculture makes it impossible for scouts to inspect every plant in every field for disease. Additionally, most diseases can spread for days to even months before symptoms become noticeable. Compounding the problem, fungicides and other control practices are universally most effective when applied to early-stage disease. Applying fungicides to well-established disease decreases the chances of successful management and increases the likelihood that resistance will emerge (41). Thus, by the time disease is discovered, it has likely already caused significant damage, and it is often too late for effective remediation.

The ability to detect early-stage disease can dramatically benefit global agriculture. Remote plant disease sensing offers monitoring at previously unachievable scales, filling gaps in space and time between labor-intensive field measurements while reducing uncertainty in downstream analyses and management decision making. At the regional level, remote sensing can support strategic use of on-the-ground expertise by preidentifying regions of likely disease to which scouts can be sent for evaluation (42–45). Proximal sensing systems deployed in-field on all-terrain vehicles (ATVs), tractors, or autonomous rovers with on-board computer vision can aid experts in monitoring larger areas (46–49). Integrating objective plant health assessments via remote sensing into existing decision support systems can improve economic injury threshold assessment while providing counterbalance to subjective human ratings without taking the ultimate decision-making away from the stakeholder (50). Spectral quantification of organic and synthetic chemical bonds in pesticides on the plant surface and in its interior could one day lead to more prescriptive fungicide application recommendations (51, 52).

Thinking more globally, forthcoming satellite systems, such as Planet Lab’s CarbonMapper (53), ESA’s Copernicus Hyperspectral Imaging Mission for the Environment (CHIME) (70), and NASA’s Surface Biology and Geology (SBG) (71, 72), will revolutionize imaging spectroscopy data availability. These systems will provide full-spectrum (400- to 2,500-nm) hyperspectral imagery at high resolution (30 m) across the entire globe. Taken as a constellation, these instruments will provide data at weekly or better intervals (without cost, in the case of CHIME and SBG) and will, for the first time, democratize the availability of such powerful data products to low- and high-income countries alike. Disease management in low-income countries is often constrained by a lack of expertise to devote to prevention, a lack of resources to devote to remediation, and a lack of qualified personnel for both these tasks (54). Compounding these challenges are unsupportive local governments, lack of equipment and support infrastructure, and preventative material export laws and costs that growers and researchers in high-income countries do not face. Plant disease sensing with these next-generation systems offers a path around these historical challenges by funneling resources and expertise from high-income countries to global regions most in need.

Plant disease sensing can help uncover biological processes driving disease patterns and cycles across scales. Zooming in to leaf-level applications, *in situ* and proximal imaging spectroscopy offers the ability to better evaluate hypotheses about plant function in response to biotic stress, pushing forward the boundary of what remote sensing may one day be capable of while adding a new dimension of study to agricultural systems biology. For example, pre- and postsymptomatic disease detection with spectral systems is now a well-established phenomenon (3, 34, 40, 55–58). However, the underlying pathosystem processes that result in distinguishable spectral features between healthy and diseased plants, as well as how common or unique these biospectral features may be across types and stages of disease, are not fully understood. Untangling the processes that contribute the most to the spectral disease phenotype will one day lead to reliable previsual disease detection and differentiation from nonbiotic stress at scale. Case studies have shown that the presymptomatic disease phenotype can differ between infections caused by both different pathogen types (5, 40, 59) and different isolates (39, 60) and can be strongly impacted by host genotype (38, 61, 62). How will these caveats affect regional and global disease monitoring? Is it possible to monitor for the specific activation of plant defenses rather than the general impact of disease on plant health to sidestep these issues? Only further research will tell.

This article has thus far focused on the benefits plant disease sensing can provide to agriculture; however, fundamental research in this domain stands to benefit multiple disciplines. Spectranomics can be thought of as a light-based DNA sequence, capturing both a plant’s current state of existence and its evolutionary history (63), thus adding a dynamic “live” dimension to multiomics studies (64). Nondestructive trait quantification with *in situ* spectroscopy allows live monitoring of infection processes previously assessable only via destructive methods (40, 62, 65). Advancements in characterizing plant status via spectranomics combined with improvements to throughput with robotics will lead to a better understanding of pathogenesis, qualitative host resistance, and, more generally, host-microbe interactions. Thinking more globally, preserving and protecting existing agroecosystems from disease is critical to conserving biodiversity in natural ecosystems and the long-term ecological health of our planet (66). Plant disease sensing can help assess regions most at risk for agri-food change disruptions and downstream impacts on food security and safety. Alert systems connected to remote sensing data could warn when vulnerable wild plant populations are under attack by pathogens, pests, and other, anthropogenic factors. Such a system also offers great opportunities for identifying novel/emerging risks to both natural and agroecosystem functions in the context of climate change and pathogen spread.

Despite its revolutionary potential, plant disease sensing remains an underdeveloped discipline, with challenges facing fundamental study and field application alike. Disease does not occur in a vacuum; in fact, it is most likely to occur in already-compromised plants with confounding abiotic and biotic stresses and is usually under active suppression attempts with chemical, cultural, and other methods of control. Multiple diseases and stressors of varying importance may therefore occur within the same pixel, and whether they can be effectively distinguished is yet unknown. This is critical to understand, because mitigation resources are limited, and endemic and invasive diseases require different urgency of response. The most destructive diseases that would be of greatest value to be able to detect are frequently under quarantine or destroy-on-discovery orders, preventing field study in the first place and forcing investigations into controlled environments. Scaling proximal and *in situ* findings is not always straightforward (67), nor is translating findings from controlled conditions to the field. Host genotype has a strong impact on diseased plant reflectance and classification accuracy (38, 61, 62), challenging universal model development. Simple indices, such as NDVI, that are widely available both commercially and from space agencies are useful for general targeting and risk assessment but have proven insufficient for diagnosis in multistress environments (68). Unlike abiotic stress, disease is the result of dynamic interactions between living organisms within ever-changing micro-, meso-, and macroclimates. The unique challenges associated with studying plant disease has yielded a discipline far behind other agricultural sensing domains, such as nutrition and water management. Above all else, there is a great need for interdisciplinary training and collaboration between plant pathologists and engineers, computer scientists, and remote sensing experts to meet these challenges and advance plant disease sensing to its fullest potential (69).

**FIG 1:**
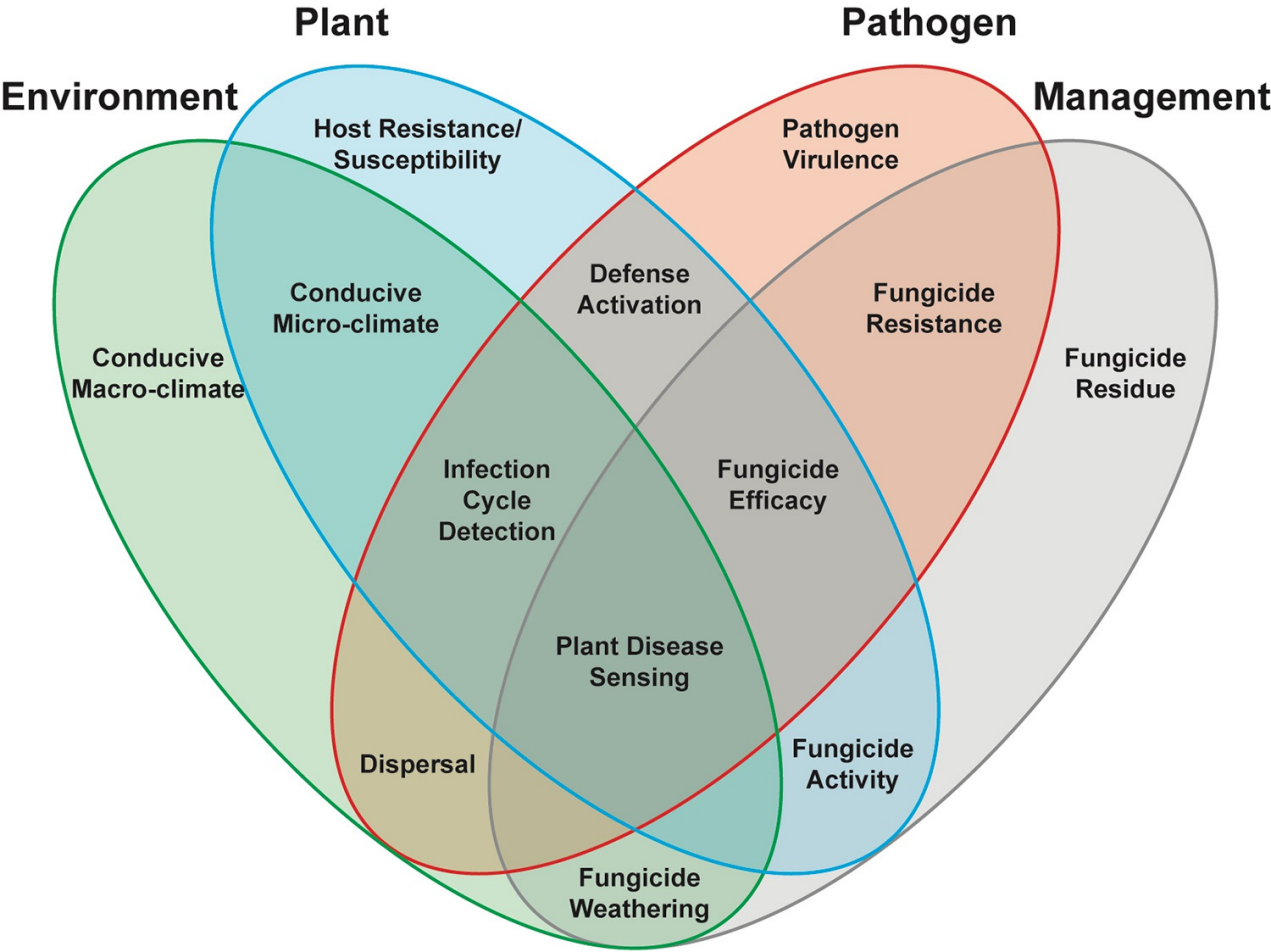
The four dimensions of plant disease sensing. Inspired by the plant disease triangle, the four dimensions of plant disease sensing encompass how the environment, host, pathogen, and human management interact with each other to yield different domains of plant disease sensing.

The Gold lab is currently the only research group worldwide wholly dedicated to studying the fundamental and applied science of plant disease sensing. Our research—funded by diverse sources ranging from NASA Earth Science Division to grape grower commodity groups—combines plant pathology, machine learning, and remote sensing to improve early disease detection and sustainable integrated management intervention. Plant pathology’s foundational theory is the disease triangle, or the concept that plant disease results from the interaction of a virulent pathogen with a susceptible host within a conducive environment. A modern interpretation of the triangle adds a fourth element—management—and acknowledges that these interactions take place in human-modified environments. In the Gold lab, we study plant disease sensing within this foundational framework (Fig. 1), in order to make progress against the previously discussed challenges. This opens up an exciting new frontier of opportunity that better harnesses time through earliest detection and management intervention to prevent crop loss and food insecurity. Oftentimes the greatest challenge in managing disease is simply finding it in the first place. We envision a world where advances in digital agriculture, remote sensing, and early intervention make this a thing of the past. Our ongoing plant disease sensing investigations include building a framework for global disease surveillance (NASA ROSES #80NSSC20K1533), asymptomatic viral disease detection (NASA ROSES #80NSSC21K1605), developing new proximal and remote sensing applications for vineyard disease management, and non-destructive fungicide activity sensing. These studies, and the future avenues of study they will lead to, will yield powerful insights into the changing climate’s impact on global disease patterns, improved security for the world’s most at-risk crops and well-being for the people that rely on them, and more mechanistic surveillance systems adaptive to not only pathosystems but also specific genotype-environment-microbe-management combinations.
